# Omega-3 fatty acids: molecular weapons against chemoresistance in breast cancer

**DOI:** 10.1186/s11658-025-00694-x

**Published:** 2025-01-25

**Authors:** Vittoria Marchio, Giuseppina Augimeri, Catia Morelli, Adele Vivacqua, Cinzia Giordano, Stefania Catalano, Diego Sisci, Ines Barone, Daniela Bonofiglio

**Affiliations:** 1 Department of Pharmacy, Health and Nutritional Sciences, University of Calabria, Arcavacata Di Rende, 87036 Cosenza, Italy; 2 Centro Sanitario, University of Calabria, Via P. Bucci, Arcavacata Di Rende (CS), 87036 Rende, Cosenza, Italy

**Keywords:** Breast cancer, Chemoresistance, Chemotherapy, Omega-3 polyunsaturated fatty acids, Natural compounds, Mediterranean diet

## Abstract

Breast cancer is the most commonly diagnosed type of cancer and the leading cause of cancer-related death in women worldwide. Highly targeted therapies have been developed for different subtypes of breast cancer, including hormone receptor (HR)-positive and human epidermal growth factor receptor 2 (HER2)-positive breast cancer. However, triple-negative breast cancer (TNBC) and metastatic breast cancer disease are primarily treated with chemotherapy, which improves disease-free and overall survival, but does not offer a curative solution for these aggressive forms of breast cancer. Moreover, the development of chemoresistance is a major cause of therapeutic failure in this neoplasia, leading to disease relapse and patient death. In addition, chemotherapy’s adverse side effects may substantially worsen health-related quality of life. Therefore, to improve the outcome of patients with breast cancer who are undergoing chemotherapy, several therapeutic options are under investigation, including the combination of chemotherapeutic drugs with natural compounds. Omega-3 (ω-3) polyunsaturated fatty acids (PUFAs), including docosahexaenoic and eicosapentaenoic acids, have drawn attention for their antitumoral properties and their preventive activities against chemotherapy-induced toxicities in breast cancer. A literature review was conducted on PubMed using keywords related to breast cancer, omega-3, chemoresistance, and chemotherapy. This review aims to provide an overview of the molecular mechanisms driving breast cancer chemoresistance, focusing on the role of ω-3 PUFAs in these recognized cellular paths and presenting current findings on the effects of ω-3 PUFAs combined with chemotherapeutic drugs in breast cancer management.

## Introduction

Breast cancer represents the most commonly diagnosed cancer and the leading cause of cancer death in women worldwide [1]. On the basis of the expression of tissue-based biomarkers, such as the hormone receptor (HR) and the epidermal growth factor 2 (ERBB2), breast cancer is classified into the following five main subtypes: luminal A (HR+/ERBB2−), luminal B (HR+/ERBB2+), normal like (HR+/ERBB2−), ERBB2+, and triple negative (TNBC) (HR−/ERBB2−), associated with different prognosis and outcome [2]. Various clinical options, including surgery, chemo-, radio-, and hormonal therapies, are available for the treatment of breast cancer, depending on the subtype, tumor histology, grade, and stage [3]. Chemotherapy represents the primary therapeutic strategy for patients with newly diagnosed HR+/ERBB2– with metastatic disease and for those facing metastatic relapse (i.e., patients refractory to endocrine therapy) or diagnosed with TNBC. In addition, chemotherapy is used to reduce tumor size before surgery or to eradicate micrometastatic disease in the neoadjuvant or postoperative systemic setting for early-stage breast cancer, respectively [3–5]. Several chemotherapeutic agents, including anthracyclines administered in single-agent or combination regimens, are currently available for breast cancer treatment. Among these, anthracyclines, such as doxorubicin, epirubicin, adriamycin, and taxanes, including paclitaxel and docetaxel, represent the chemotherapeutic drugs used most widely in routine clinical practice. Although chemotherapy improves breast cancer survival, it carries several toxicities, including hair loss, bone marrow suppression, neuropathies, gastrointestinal and skin disorders, which worsen physical–psychological health and quality of life [6]. Moreover, chemotherapy efficacy decreases during treatment, owing to chemoresistance development, leading to disease relapse and patient death [7]. Recently, cyclin-dependent kinase (CDK) 4/6, polyADP-ribose polymerase (PARP), and phosphoinositide 3-kinase (PI3K) inhibitors as well as immunotherapy have been investigated for TNBC treatment to supplant traditional chemotherapy [8]. However, the results are still unsatisfactory and research is ongoing to find new efficient drugs showing minimal side effects or able to prevent/counteract the development of chemoresistance in breast cancer.

Omega-3 polyunsaturated fatty acids (ω-3 PUFAs), including docosahexaenoic (DHA) and eicosapentaenoic (EPA) acids, have been studied as supportive therapeutic options for the treatment of several cancers, including breast cancer [9–11]. Indeed, ω-3 PUFAs, found in different foods of the Mediterranean Diet, showed antitumoral effects in preclinical breast cancer models [12–14] and ameliorated the side effects of chemotherapy [8], improving clinical outcomes. ω-3 PUFAs can also impact breast cancer development and progression through their conversion into endogenous metabolites, including the *N*-acyl conjugates, which showed higher activity compared with parent compounds in several reports [15, 16].

The present study aims to review research on the effects of ω-3 PUFAs combined with chemotherapeutic drugs in the treatment of breast cancer, underlining their role in modulating breast cancer chemoresistance. First, we summarize updated information regarding the molecular mechanisms responsible for chemoresistance in breast cancer. We then report compelling evidence on the role of ω-3 PUFAs in counteracting breast cancer progression and drug resistance. Finally, we present the current knowledge about the effects of combined treatment with ω-3 PUFAs and chemotherapeutic drugs in breast cancer from in vitro, in vivo, and clinical studies.

## Molecular mechanisms of chemoresistance in breast cancer

Two different types of chemoresistance have been described, viz. intrinsic and extrinsic chemoresistance. Tumors showing intrinsic resistance are not responsive to therapeutic treatment even before the initial administration of the drug. This type of chemoresistance, often associated with inherent genetic mutations within tumors, high cancer cell population heterogeneity, or pharmacological factors, such as inadequate drug concentration at the tumor site, can be avoided by performing genomic or biochemical analysis before the beginning of drug treatment. Extrinsic chemoresistance occurs after an initial pharmacological response and depends on molecular changes in the drug targets or the components of the tumor microenvironment [17, 18]. A brief overview of the major drug chemoresistance mechanisms in breast cancer is discussed in the next section.

### Expression of efflux transporters and chemoresistance

The most common mechanism of chemoresistance is associated with overexpression of adenosine triphosphate (ATP)-binding cassette (ABC) transporters on the membrane of cancer cells. ABC transporters are transmembrane proteins involved in the transport of a wide spectrum of substrates across the membrane through the hydrolysis of ATP. In cancer cells, ABC transporters reduce the intracellular drug accumulation [19, 20]. Several ABC transporters have been described, including the multidrug-resistant protein-1 (MRP1), the P-glycoprotein (P-gp), and the breast cancer resistance protein (BCRP) [20], being associated with shorter survival in patients with breast cancer [21, 22].

### DNA damage and chemoresistance

In normal cells, DNA damage is repaired by the activation of DNA damage repair (DDR) pathways to maintain genomic stability, ensuring the accurate transmission of the genetic information to the next generation. Chemotherapy induces DNA damage, including double-strand breaks (DSBs), which are repaired through homologous recombination (HR) or nonhomologous end-joining (NHEJ) pathways. Increased levels of the kinase ataxia telangiectasia mutated (ATM), which mediate the HR pathway, were found in chemoresistant breast cancer cells [23], and cisplatin treatment was associated with increased expression of the DSB repair protein RAD51, inducing chemoresistance in breast cancer cells [24]. Accordingly, RAD51 inhibition sensitized chemoresistant breast cancer cells to doxorubicin and docetaxel [25] and increased the sensitivity of breast cancer cells to adriamycin and cisplatin [26].

### Evasion of apoptosis and chemoresistance

Cells trigger apoptosis, or programmed cell death, through the activation of intrinsic or extrinsic apoptosis pathways, to maintain tissue homeostasis and eliminate damaged cells. The extrinsic pathway is activated by the binding of ligands to transmembrane death receptors, including the members of the tumor necrosis factor (TNF) receptor family, which leads to the formation of the death-inducing signaling complex and the activation of caspase 8. The intrinsic pathway, also known as the mitochondrial pathway, is regulated by the B-cell lymphoma 2 (Bcl-2) protein family, which includes proteins with pro-apoptotic (Bax, Bak, and Bcl-xS) or anti-apoptotic (Bcl-2, Bcl-xLand Mcl-1) activities [27] Pro-apoptotic proteins induce the release of cytochrome *c *from the mitochondria to the cytoplasm, triggering the formation of the apoptosome and the activation of caspase 9. In contrast, anti-apoptotic proteins inhibit apoptosis, maintaining the mitochondrial integrity. The balance between apoptotic and anti-apoptotic signals is important to determine the cell’s fate. In resistant cancer cells, the balance between apoptotic and anti-apoptotic signals is disrupted, leading to the evasion of apoptosis, one of the primary hallmarks of cancer. Altered death receptor signaling was found to trigger epithelial-to-mesenchymal transition (EMT) and a multidrug resistant phenotype [28]. At the molecular level, chemosensitive MCF-7 breast cancer cells resistant to TNF exhibited reduced expression of TNF receptor 1 (TNFR1) and TNFR1-associated death domain protein (TRAD), which blocked extrinsic apoptosis and increased the nuclear factor-kappa B (NF-κB) survival pathway. In addition, these cells showed decreased sensitivity to several drugs, including doxorubicin, taxol, and etoposide [28]. Breast cancer cells can also acquire resistance to paclitaxel by switching from apoptosis to autophagy, which results in downregulation of the mammalian target of rapamycin (mTOR) pathway along with an increased ability of breast cancer cells to survive under stress conditions [29]. Therefore, certain proteins involved in apoptosis are being investigated as potential targets for breast cancer treatment [30]. For instance, preclinical studies indicate that Bcl-2 inhibitors such as ABT-199 can sensitize TNBC cells to doxorubicin, offering a promising strategy to overcome chemoresistance [31].

### Activation of signaling pathways related to tumorigenesis and chemoresistance

The hyperactivation of several signaling pathways that regulate breast cancer cell survival, growth, and invasion is involved in the development of breast cancer chemoresistance [32]. Stevens and collaborators found that chemoresistance is mediated by the activation of the Janus kinase (JAK)-2/signal transducer and activator of transcription (STAT) 3 and the cyclic adenosine 3′,5′-monophosphate (cAMP)/protein kinase A (PKA) signaling pathways as well as changes in the cell phenotype in inflammatory breast cancer [33]. Receptor tyrosine kinases (RTKs), such as HER-2, are also key factors involved in breast cancer chemoresistance through activation of the phosphoinositide 3-LKinase (PI3K)/protein kinase (AKT) signaling pathway. HER-2 amplification was associated with resistance to different chemotherapeutic drug, including cyclophosphamide, methotrexate, and epirubicin [34].

### Hypoxia and chemoresistance

Hypoxia is a common condition in solid tumors owing to inadequate oxygen supply at the tumor site, which results in rapid cell proliferation and impaired blood flow and represents a negative prognostic marker in cancer [35]. Low intratumoral levels of oxygen are associated with a more aggressive breast cancer phenotype characterized by metastatic properties and reduced sensitivity to chemotherapy [36]. Many biological changes associated with tumor hypoxia are mediated by the induction of hypoxia-inducible factors (HIFs), including HIF-1α [37–40]. Increased tumor HIF-1α levels were associated with a decreased overall response to epirubicin in patients with breast cancer [41] and with an overexpression of several genes sustaining angiogenesis, and drug resistance [42]. Moreover, HIF-1α activation promotes metabolic reprogramming [43], and breast cancer stem cell (BCSC) enrichment [44], which support chemoresistance.

### Metabolic reprogramming

Metabolic reprogramming is considered to be one of the hallmarks of cancer and is recognized as a mechanism of chemoresistance. Indeed, cancer cells require changes in the metabolism to meet the needs of ATP production required for the high rate of cell proliferation. Moreover, it has been found that deregulated expression of metabolic genes as well as activation of metabolic pathways, such as glucose and lipid metabolisms, contribute to reduce the efficacy of chemotherapeutic drugs [45, 46]. It has been widely demonstrated that cancer cells rely on aerobic glycolysis for energy production (the Warburg effect), which contributes to chemoresistance through epigenetic and genetic regulation of survival/death pathways [47]. Not only glucose metabolism but also lipid and amino acid metabolisms are reprogrammed in cancer cells [48]. Indeed, using metabolomic approaches, it has been observed that breast cancer cell lines modified the acetate, lactate, and phosphocholine metabolism on the basis of their molecular subtype upon treatment with antitumoral drugs, including tamoxifen, cisplatin, and doxorubicin [49]. Similarly, other authors have found an altered metabolism of nitrogenous bases, glucose, and lipids in breast cancer cell lines resistant to adriamycin [50, 51]. Altered lipid metabolism plays a crucial role in the development of chemoresistance since adipocytes represent one of the major cellular components of breast cancer tissue. Indeed, the high rate of lipid metabolism, including the degradation of fatty acids, cholesterol, and phospholipids, provides an energy source for cancer cell invasion and migration, leading to the development of chemoresistance [52, 53]. Thus, inhibiting lipid metabolism using drugs such as statins, the inhibitor of carnitine palmitoyltransferase I (CPT1) etomoxir and the inhibitor of fatty acid synthase (FASN) TVB-2640 is emerging as a new strategy to counteract breast cancer chemoresistance [54–57]. For instance, it has been observed that TVB-2640 reverses the resistance to taxane in breast cancer in a phase I clinical trial [56].

### Remodeling of the tumor microenvironment and chemoresistance

The breast cancer tumor microenvironment consists of stromal cells, such as cancer-associated fibroblasts (CAFs), tumor-associated macrophages (TAMs), cancer-associated adipocytes (CAAs), mesenchymal stem cells (MSCs), BCSCs, endothelial cells (ECs), and immune cells, and acellular components, including the extracellular matrix (ECM), soluble factors, and extracellular vesicles (EVs). Their reciprocal interaction promotes a permissive milieu that reduces the sensitivity of breast cancer cells to chemotherapeutic drugs [58].

#### Cellular components within tumor microenvironment

CAFs represent a major component of the tumor microenvironment, involved in tumor growth, metastasis, and chemoresistance. CAFs secrete soluble factors, including the transforming growth factor (TGF)-β, which promotes EMT and chemoresistance by MAPK p44/42 signaling activation [59]. Moreover, Su and collaborators described a subpopulation of CAFs, characterized by CD10 and GPR77 expression, able to sustain chemoresistance through the secretion of interleukin (IL)-6 and IL-8 [60].

TAMs support breast cancer progression, metastasis, and therapeutic responses, and their abundance in breast cancer is considered to be a negative prognostic factor [61]. Paclitaxel treatment induced the expression of colony-stimulating factor (CSF)-1 in tissues from chemoresistant breast cancer xenografts, which stimulated the recruitment of TAMs, while CSF-1 signaling inhibition reduced TAM recruitment and restored paclitaxel antitumoral effects [62]. In addition, it was demonstrated that TAM-secreted IL-10 was responsible for paclitaxel resistance in breast cancer cells, leading to upregulated Bcl-2 expression and STAT3 signaling activation [63].

Obesity has an intricate relationship with both breast cancer occurrence and the clinical behavior of the established malignancy. The obese setting offers a distinctive adipose tumor microenvironment that, along with systemic alterations, fosters breast cancer development and progression [64, 65]. In the tumor microenvironment, CAAs secrete multiple factors, including leptin, promoting breast cancer aggressiveness and chemoresistance [66–70]. Moreover, CAAs increased drug efflux from breast cancer cells, mediated by enhanced expression of efflux transporters [71, 72].

In addition, MSCs are pluripotent cells that can differentiate into different cell types, including adipocytes, osteoblasts, chondrocytes, myoblasts, adipocytes, and fibroblasts, which modulate breast cancer progression and chemoresistance within the breast tumor microenvironment. In particular, MSCs produce C–X–C motif chemokine ligand 1 (CXCL1), inducing doxorubicin resistance in TNBC cells, through decreased miRNA 106a levels and increased ABCG2 transporter expression [73]. Similarly, breast cancer cells secreted IL-6 that stimulated MSC recruitment from the bone marrow to the tumor site, and the subsequent release of CXCL17, thereby supporting stemness and chemoresistance [74]. MSCs can also support breast cancer chemoresistance through a physical interaction with breast cancer cells. In this context, we found that MSCs can be engulfed by breast cancer cells, generating a hybrid cell population with reduced sensitivity to doxorubicin and paclitaxel [75].

There is increasing evidence that BCSCs contribute to chemoresistance through increased aldehyde dehydrogenase (ALDH1) expression [76, 77], upregulation of the ABCG-2 transporter [78], and deregulation of apoptosis [79]. Moreover, interaction among breast cancer cells, ECs, and myeloid cells promotes chemoresistance via chemokine (C–X–C motif) ligand 1/2 chemokine networks [80]. In particular, chemotherapy induced the secretion of TNFα by ECs, increasing the production of CXCL1/2 by breast cancer cells. In turn, breast cancer cells increased S100A8/9 expression by myeloid cells, supporting breast cancer cell survival [80].

#### Acellular components within the tumor microenvironment

By regulating cell–cell and cell–matrix interactions, the ECM strongly supports chemoresistance. ECM proteins, such as β1 integrin and fibronectin, mediated breast cancer cell adhesion to ECM and reduced the efficacy of paclitaxel, vincristine [81], and docetaxel [82]. On the other hand, by activating several pathways in breast cancer cells, including the JNK pathway, chemotherapy stimulated the secretion of different matrix proteins (i.e., osteopontin, tenascin C, the B-lymphoma Mo-MLV insertion region 1, and phosphatase and tensin homolog), thereby facilitating breast cancer progression and chemoresistance [83].

EVs are also important players in chemoresistance owing to their unique cargo of nucleic acids, proteins, lipids, and metabolites, which they transport across cells [84]. EVs can support breast cancer chemoresistance through different mechanisms, including drug efflux, transfer of ABC transporters, prosurvival signaling molecules, and drug-metabolizing enzymes from resistant to nonresistant cells [85, 86]. Recently, we demonstrated that EVs released by adipocytes increased breast cancer aggressiveness via HIF-1α [85], which as mentioned above, is strictly related to breast cancer chemoresistance. Additionally, EVs from obese patients with breast cancer exhibited lower let-7a levels, which correlated with higher tumor grade and poorer survival, proposing novel biomarkers for obesity-related breast cancer [87].

### Schematic summary of the main mechanisms of chemoresistance in breast cancer

**Fig. 1:**
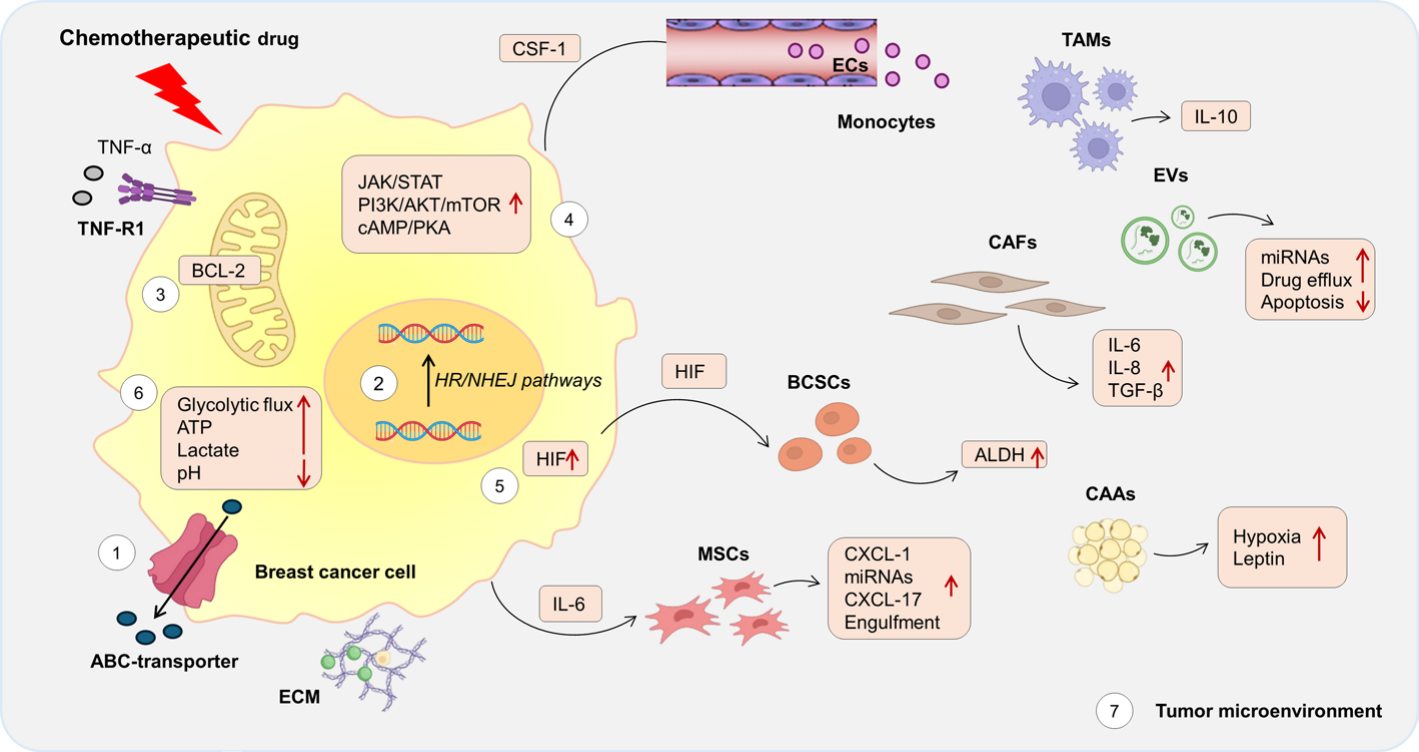
Main mechanisms involved in drug resistance. Upon treatment with chemotherapeutic drugs, breast cancer cells can develop chemoresistance by: inducing drug efflux via the ATP-binding cassette (ABC) transporters (1), enhancing DNA repair through homologous recombination (HR) and nonhomologous end joining (NHEJ) pathways (2), reducing activation of intrinsic and extrinsic apoptosis pathways (3), activating prosurvival signaling pathways (4), increasing the expression of hypoxia-inducible factor (HIF) in a hypoxic microenvironment (5), reprogramming metabolism (6), remodeling the tumor microenvironment (7). CSF, colony-stimulating factor; IL, interleukin; TGF, transforming growth factor; CXCL, C–X–C motif chemokine ligand; miRNA, microRNA; TNF, tumor necrosis factor; TNFR, tumor necrosis receptor; ALDH, aldehyde dehydrogenase; CAFs, cancer-associated fibroblasts; TAMs, tumor-associated macrophages; BCSCs, breast cancer stem cells; EVs, extracellular vesicles; MSCs, mesenchymal stem cells; ECM, extracellular matrix; ABC-transporters, ATP-binding cassette transporters; HR, hormone receptors; NHEJ, nonhomologous end joining; HIF, hypoxia-inducible factors; Bcl, B-cell lymphoma; JAK, Janus kinase; STAT, signal transducer and activator of transcription; PI3K, phosphoinositide 3-LKinase; AKT, protein kinase B; mTOR, mammalian target of rapamycin; NF-kB, nuclear factor-kappa B; cAMP, cyclic adenosine 3′,5′-monophosphate; PKA, protein kinase A. Figure created with PowerPoint by Microsoft Office 365

On the basis of the above-described observations, several molecular mechanisms for chemoresistance in breast cancer have been proposed. These mechanisms are depicted schematically in Fig. 1.

## Impact of ω-3 PUFAs in modulating the molecular mechanisms associated with chemoresistance in breast cancer

**Fig. 2:**
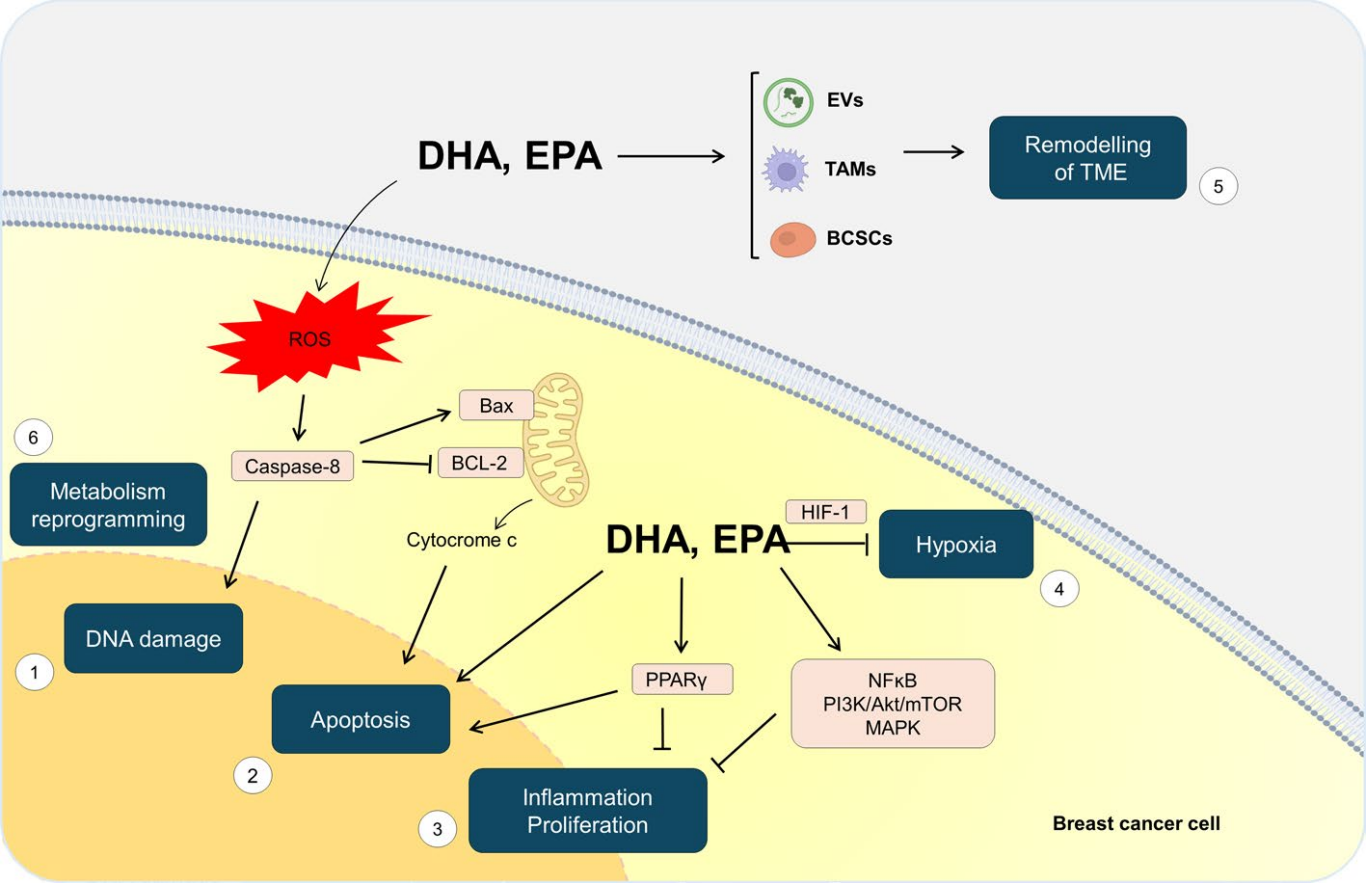
Effects of DHA and EPA in modulating the molecular mechanisms of chemoresistance in breast cancer. DHA and EPA may impact chemoresistance by: enhancing reactive species oxygen (ROS) production and inducing DNA damage and apoptosis (1); inhibiting the expression of hypoxia-inducible factor (HIF)-1α, and reducing hypoxia (2); decreasing the activation of signaling pathways involved in inflammation and proliferation (3); activating PPARγ, which promoted apoptosis (4); modulating tumor microenvironment (5); reprogramming metabolism (6). Bcl, B-cell lymphoma; PI3K, phosphoinositide 3-LKinase; AKT, protein kinase B; mTOR, mammalian target of rapamycin; NF-kB, nuclear factor-kappa B; MAPK, activated protein kinase. Figure created with PowerPoint by Microsoft Office 365

ω-3 PUFAs, including DHA and EPA, are essential nutrients that exert multiple health benefits. In the human body, DHA and EPA can be biosynthesized from α-linolenic acid (ALA), a short-chain essential fatty acid, through enzymatic elongation and desaturation reactions that primarily occur in the liver. Epidemiological studies and controlled trials indicate that plant- and sea-derived PUFAs are likely to be important mediators of the protection provided by the traditional Mediterranean Diet. Indeed, ω-3 PUFAs contribute significantly to the structural integrity of cell membranes, regulating various downstream cellular functions and preventing the development of a wide spectrum of diseases, including cardiovascular diseases [88], diabetes mellitus [89], depression, various mental illnesses [89], age-related cognitive decline [90], and various types of cancer, such as breast cancer [91]. In particular, it has been demonstrated that ω-3 PUFAs can modify the physical–chemical properties of membranes, determining changes in their lysis tension, water permeability, and elasticity [92, 93]. Moreover, ω-3 PUFAs lower the cholesterol levels in membranes [94], which is important for cell membrane fluidity. ω-3 PUFAs have been also found to regulate the activity of membrane receptors, including channel and G-protein coupled membrane receptors, through which they can affect the activation of downstream signaling pathways [95, 96]. The ability of ω-3 PUFAs to impact several pathways associated with chemoresistance in breast cancer has been widely investigated over time. Here, we provide a review of the current knowledge on this research topic and present a schematic representation of the underlying molecular mechanisms (Fig. 2).

### ω-3 PUFAs and efflux transporters in breast cancer

Although various ABC transporters involved in multidrug resistance (MDR) are sensitive to the lipid plasma membrane composition, to the best of the authors’ knowledge, there are no studies investigating the role of ω-3 PUFAs in modulating the expression of ABC transporters in breast cancer. However, it was found that ω-3 PUFAs reduced the total membrane- and lipid-raft-associated Pgp and MRP1, increasing the sensitivity to doxorubicin and irinotecan in colon cancer HT9/MDR cells [97]. Consistent with these data, other authors reported that ω-3 PUFAs decreased the expression of Pgp and MRP1 and their activity, enhancing the antitumor effects of several chemotherapeutic drugs in detergent-resistant membranes of human chemosensitive colon cancer cells [98]. These results provide the rationale to further explore the potential role of ω-3 PUFAs in counteracting the development of chemoresistance by modulating the expression of efflux transporters in breast cancer.

### ω-3 PUFAs and DNA damage in breast cancer

Several studies have demonstrated that ω-3 PUFAs induce DNA damage in cancer cells, suppressing tumor development and enhancing the effectiveness of chemotherapeutic drugs. DHA was found to inhibit DNA synthesis, triggering apoptosis in MCF-7 breast cancer cells. In particular, DHA reduced DNA synthesis by 50%, while 90% of breast cancer cells retained viability, indicating that the inhibition of DNA synthesis could precede cell viability decrease [99]. The authors speculated that DHA treatment induced intracellular reactive oxygen species (ROS) accumulation and protein nitration in MCF-7 breast cancer cells, which resulted in DNA damage and induction of caspase-8-dependent apoptosis [99]. In line with these in vitro results, increased levels of 3-nitrotyrosine, a marker used for protein nitration and cellular oxidative stress, were found in tumors from mice fed with fish oil compared with control-diet-fed animals [99]. Manna and collaborators investigated the effects of fish oil, enriched with ω-3 PUFAs, against carcinogen-induced DNA damage in animal models, and found reduced mammary tumorigenesis, cell proliferation, and angiogenesis in rats fed with fish oil. In addition, they observed lower levels of 8-hydroxy-20-deoxyguanosine, a marker of DNA damage, in mammary tissue of rats fed with fish oil compared with the carcinogen control group, suggesting the ability of ω-3 PUFAs to protect against carcinogen-induced DNA damage [100].

### ω-3 PUFAs and apoptosis in breast cancer

Both DHA and EPA have been found to activate apoptosis in various breast cancer cell lines, including MCF-7, MDA-MB-231, and MDA-MB-435S breast cancer cells, in a concentration-dependent manner [101]. Accordingly, a 5% fish-oil-supplemented diet enriched with ω-3 PUFAs for 30 days significantly diminished tumor growth and increased apoptosis in mammary-tumor-bearing mice [102]. ω-3 PUFAs also exerted pro-apoptotic effects in breast cancer, targeting different apoptotic molecules. In particular, DHA was shown to increase the expression of the pro-apoptotic protein Bax and reduce the protein expression of Bcl-2. Moreover, it induced the mitochondria release of cytochrome *c* and the activation of caspase 8, 9, and 3, triggering cell apoptosis [103]. Fatty acids can be taken up by mammary cells either via internalized low-density lipoprotein (LDL) or through a complex with albumin. Edwards and collaborators reported that ω-3 PUFAs conjugated with LDL induced apoptosis in MCF-7 breast cancer cells, increasing the cleavage of PARP and reducing the procaspase 3 protein expression [104]. In contrast, only DHA conjugated with albumin, but not EPA, induced apoptosis in MCF-7 breast cancer cells through upregulation of the tumor suppressor syndecan-1 (SDC-1) in a peroxisome proliferator-activated receptor (PPAR) γ-dependent manner [105]. Interestingly, other metabolites of DHA and EPA might exert anti-apoptotic effects in breast cancer cells, acting as PPARγ ligands. In this context, we previously demonstrated that the conjugates of DHA and EPA with dopamine induced antiproliferative effects in MCF-7, SKBR3, and MDA-MB-231 breast cancer cells through activation of PPARγ, which upregulated expression of Beclin-1. This mechanism enhanced the autophagic flux and subsequently triggered the apoptotic cascade [106]. Similarly, the conjugates of ω-3 PUFAs with ethanolamine induced autophagy in MCF-7 breast cancer cells through activation of PPARγ [107]. Another mechanism by which ω-3 PUFAs mediated apoptosis relies on the regulation of different antioxidant enzyme activity. Geng and collaborators demonstrated that DHA increased the activity of total superoxide dismutase, catalase, and glutathione‑peroxidase in human malignant breast cancer tissues, inducing apoptosis [108]. Besides its involvement in apoptosis, DHA also promoted pyroptosis, an inflammation-related cell death. Indeed, DHA stimulated the translocation of NF-κB into the nucleus, decreased the expression of procaspase-1, increased the IL-1β secretion as well as the expression of the inflammasome adapter protein ASC, inducing the pore membrane formation and the cell death in TNBC cells [109].

### ω-3 PUFAs and signaling pathways involved in chemoresistance

Several studies have highlighted the ability of ω-3 PUFAs to inhibit the activation of various signaling pathways involved in breast cancer chemoresistance. Treatment of MDA-MB-231 breast cancer cells with EPA and DHA leads to the inhibition of the survival Akt/NFκB signaling pathway [110]. Similarly, Ghosh-Choudhury and collaborators demonstrated that dietary fish oil significantly suppressed PI3K activity in breast cancer, resulting in reduced phosphorylation of AKT and NFκB p65 subunit as well as expression of anti-apoptotic proteins [111]. Moreover, both DHA and EPA suppressed NFκB transcriptional activation, thus mitigating the transcription of pro-apoptotic genes [111]. The conjugates of ω-3 PUFAs with ethanolamine were also shown to decrease the activation of the PI3K/Akt/mTOR signaling pathway in MCF-7 breast cancer cells, inducing cell death [107]. Xue and collaborators showed that ω-3 PUFAs significantly reduced the β-catenin levels and the expression of the Wingless-related integration site (Wnt)/β-catenin targets genes, such as c-myc and cyclin D1, in mammary tumors from female outbred Babl/c mice injected with 4T1 mouse breast cancer cells [102]. DHA was found to reduce cell proliferation excluding the epidermal growth factor receptor (EGFR) from the lipid rafts localized in the cell membrane, which resulted in decreased activation of Ras and the MAPK signaling pathways [112].

### ω-3 PUFAs and hypoxia in breast cancer

To date, only DHA has been explored as a molecule able to modulate hypoxia in breast cancer. DHA was shown to reduce the expression of HIF-1α and its target genes, such as the glucose transporter 1 and lactate dehydrogenase, in BT-474 and MDA-MB-231 breast cancer cells. This mechanism is associated with decreased glycolytic activity and mitochondrial respiration, which sustain cancer progression [113]. Other authors have demonstrated that DHA reduced the expression of HIF-1α and several angiogenic factors, including TGF-β, Snail-1 and -2, and vascular endothelial growth factor receptor (VEGFR) in MDA-MB-231 and BT-474 breast cancer cells and in EVs derived from these lines under hypoxic conditions, suggesting that DHA can inhibit breast cancer progression, also inducing anti-angiogenic effects [114].

### ω-3 PUFAs and metabolism reprogramming

It has been observed that ω-3 PUFAs induced ferroptosis in TNBC cells by regulating the fatty acid binding protein 5 (FABP5), which is a lipid sensor. In particular, the authors demonstrated that the downregulation of FABP5 reduced ferroptosis upon treatment with DHA in TNBC cells. In addition, mutation in FABP5 decreased the maximal mitochondrial respiration as well as the oxygen consumption and ROS production in DHA-induced ferroptosis [115]. DHA treatment decreased the extracellular acidification rate (ECAR) and the oxygen consumption rate (OCR) in BT-474 and MDA-MB-231 breast cancer cells, whereas it did not induce metabolic changes in nontumorigenic MCF-10A breast epithelial cells [116]. Similarly, we have previously demonstrated that the conjugate of DHA with ethanolamine, DHEA, reduced both oxygen consumption and ATP production, conferring reduced survival advantages on breast cancer cells [117]. Moreover, it has been found that DHA enhanced the function of LKB1, the target of AMPK, inhibiting the glycolytic enzymes and the mTOR signaling in breast cancer cell lines [118]. Treatment of BCSCs with ω-3 PUFAs downregulated the expression of lipogenic enzymes, including stearoyl-CoA desaturase 1 (SCD1) and fatty acids synthase, thus reducing BCSC self-renewal and growth [84]. Bobin-Dubigeon et al. showed that ω-3 PUFAs affected breast cancer severity depending on the type of lipoprotein carrying these molecules. Using sera from patients with breast cancer categorized on the basis of HR status and the level of the Ki67 proliferation marker, they investigated the concentration of EPA and DHA in HDL and non-HDL, which are the two groups of circulating lipoproteins used in clinical and epidemiological studies. They found a significant higher ratio of HDL EPA/apolipoprotein B non-HDL and EPA/HDL EPA in the sera from Ki67−/HR− patients compared with Ki67+/HR− patients, suggesting a protective role of EPA carried by non-HDL particles in this subtype of breast cancer [119].

### ω-3 PUFAs and the tumor microenvironment

Limited studies have been conducted to investigate the effects of ω-3 PUFAs in remodeling tumor microenvironment. ω-3 PUFAs have been implicated in the regulation of breast cancer stemness. It has been demonstrated that ω-3 PUFAs interact with BCSCs, reducing their proliferation, viability, and capability to form tumorspheres [120]. Luo et al. demonstrated that DHA and EPA inhibited self-renewal and growth of BCSCs using two different experimental models: mammospheres derived from MCF‐7 and human mammary epithelial (HMLE)-Twist-ER breast cancer cells treated with tamoxifen to induce the EMT phenotype. Moreover, the authors demonstrated that EPA and DHA supplementation reduced tumor growth in a mouse xenograft model using MCF‐7‐CSC cells [13]. It was observed that DHA reduced the percentage of ALDH + BCSCs and mammosphere formation in the MDA-MB-231 breast cancer line, negatively modulating cancer stem-like features [121]. In addition, in a co-culture system, we found that TNBC cells exposed to the conjugate of ω-3 PUFAs with ethanolamine suppressed macrophage recruitment and cell viability [122]. More recently, we demonstrated that the conjugate of ω-3 PUFAs reduced the expression of genes related to the TAM phenotype, including IL-6, matrix metalloproteinase (MMP)-9, VEGF, IL-10, and monocyte chemoattractant protein (MCP)-1 [117]. In addition, we also showed that the conjugate of DHA with ethanolamine and serotonin reduced the secretion of IL-6 and IL-1 receptor antagonist by breast TAMs, attenuating their malignant phenotype [122]. DHA also exerted anti-angiogenic actions through the secretion of exosomes enriched with miRNA, including let-7a, miR-23b, miR-27a/b, miR-21, and miR-320b, which reduced the secretion of pro-angiogenic factors by endothelial cells [123]. Maralbashi and collaborators demonstrated that DHA reduced mTOR levels and enhanced the expression of its targeted miRNA, miR-214, in exosomes released by TNBC cells in normoxic and hypoxic conditions. The authors speculated that DHA exerted antitumoral effects in breast cancer, modulating their exosome content [124].

## Effects of ω-3 PUFAs in modulating the efficacy of chemotherapeutic drugs

Several studies have demonstrated that ω-3 PUFAs can enhance the effectiveness of chemotherapy drugs, overcoming the development of drug resistance [125]. Here, the in vitro, in vivo, and clinical evidence on the effects of combined treatment with ω-3 PUFAs and chemotherapeutic drugs in breast cancer are described.

### In vitro studies

ω-3 PUFAs have been demonstrated to induce chemosensitization through diverse mechanisms. Rushing and collaborators demonstrated that DHA and EPA sensitized TNBC cells to doxorubicin by altering the metabolism of breast cancer cells. In particular, ω-3 PUFAs induced changes in the amino acid metabolism and fatty acid oxidation, increasing the sensitivity of breast cancer cells to chemotherapy [126]. Mahéo and collaborators observed that DHA increased the levels of malondialdehyde, a byproduct of lipid peroxidation, which enhanced doxorubicin toxicity in breast cancer cells. Moreover, DHA increased the glutathione levels, reducing the utilization by its consuming enzymes, such as glutathione peroxidase or glutathione *S*-transferase, boosting the chemosensitivity of breast cancer cells to doxorubicin [127]. ω-3 PUFAs also enhanced the cytotoxic effects of doxorubicin, inducing DNA oxidative stress [128]. Besides oxidative stress, ω-3 PUFAs exhibited chemosensitizing effects in breast cancer through alteration of the membrane lipid composition within the lipid rafts in terms of the content and function of transmembrane proteins, such as receptors, growth factors, and ABC transporters [129]. Chavin and collaborators reported that DHA decreased the activation of ERK and AKT pathways induced by docetaxel, enhancing its toxicity in MDA-MB-231 breast cancer cells [130]. Furthermore, Crovella and collaborators investigated the effects of a combination of DHA with doxorubicin in chemotherapy-resistant breast cancer cells, demonstrating that DHA enhanced breast cancer sensitivity to doxorubicin via G2/M phase cell cycle arrest, apoptosis, and lipid peroxidation [131]. In addition, DHA downregulated the expression of drug-efflux-regulating genes, such as P-gp and TG2 [131]. Although still limited, these experimental studies collectively underscore the multifaceted roles of ω-3 PUFAs in enhancing the efficacy of chemotherapy drugs, providing valuable insights into sensitization mechanisms and potential strategies to overcome drug resistance in breast cancer cells.

### In vivo studies

In animal models, dietary supplementation with ω-3 PUFAs improved the efficacy of chemotherapeutic drugs in breast cancer [132]. Newell and collaborators showed that DHA improves the efficacy of doxorubicin in female nu/nu mice bearing MDA-MB-231 tumors. In particular, mice were randomly assigned to a diet containing 20 ± 2.8 g DHA/100 g diet, with or without injections 2 times/week of 5 mg doxorubicin/kg for 4 weeks. Their findings demonstrated that DHA reduced the tumor size by promoting apoptosis and overexpressing critical cell cycle genes, including caspase-10, BH3 interacting domain death agonist (BID), and CD95 compared with the control group [133]. In a separate study, the same research team employed a similar experimental setup to investigate the effect of a diet supplemented with DHA (3.9% weight/weight of total fat) in combination with docetaxel in two different drug-resistant patient-derived xenografts (PDX): (i) MAXF574, a poorly differentiated, and well-vascularized PDX; (ii) MAXF401, a moderately differentiated and poorly vascularized model. Mice bearing xenografts that received the DHA-supplemented diet and docetaxel treatment exhibited a reduction in tumor weight. In addition, higher expression of the pro-apoptotic proteins Ripk1 and BID and lower levels of the anti-apoptotic proteins Bcl-2 and PARP along with decreased expression of the proliferation marker Ki67 and the cell-cycle regulator protein Survivin were found in the tumors from mice fed with DHA compared with the control group [133]. Colas and collaborators induced mammary tumorigenesis in Sprague–Dawley rats receiving supplementation with palm oil or DHA and treated with epirubicin to investigate the role of DHA in modulating tumorigenesis before and during the chemotherapeutic setting [134]. Although DHA was not able to reduce the incidence of tumor burden compared with the control group, it induced a significant mammary tumor regression after the beginning of chemotherapy. Indeed, after treatment with epirubicin, DHA reduced the mammary tumor growth by 45% compared with control. In addition, DHA reduced the power Doppler vascularity index (PDI) by 35%, indicating diminished tumor vascularization associated with this treatment [134]. Consistent with these results, Colas and collaborators confirmed that supplementation with DHA sensitizes mammary tumors to epirubicin, enhancing its antitumor activity in female Sprague–Dawley rats bearing chemically induced breast tumors. In particular, after inducing mammary tumorigenesis by *N*-methylnitrosourea administration, rats were fed either a diet supplemented with DHA or palm oil as a control. When tumors reached 1.5 cm^2^, rats were treated with epirubicin 2.5 mg/kg for 6 weeks. Before the initiation of chemotherapy, DHA decreased the mean PDI value in mammary tumors, suggesting its ability to lower vascularization, but it did not reduce the tumor growth rate. However, a significantly reduced tumor growth was observed in rats fed with DHA compared with control after epirubicin treatment, suggesting that DHA may increase the response of mammary tumors to chemotherapy [134]. Interestingly, the ability of DHA to increase the sensitization of chemotherapeutic drugs is selective to breast cancer cells, while it does not affect normal mammary cell behavior. Indeed, using the model of chemically induced mammary tumors proposed by Colas and collaborators, Hajjaji and collaborators observed that rats fed with DHA and treated with epirubicin showed increased lipid hydroperoxides in tumors, but not in normal tissue, suggesting that the selective toxicity of DHA supplementation in tumors during chemotherapy depends on the differential induction of oxidative stress in tumors compared with other tissues [135]. Moreover, incorporating 3% fish oil concentrate (FOC) into the diet of mice bearing mammary tumors increased ω-3 PUFA levels in tumor and liver cell membranes compared with a corn oil diet, enhancing the ability of doxorubicin to reduce breast tumor growth, without increasing its toxicity compared with corn oil diet. In particular, FOC diet altered the antioxidant enzyme balance in the mammary tumors, leading to increased oxidative stress and reduced tumor growth rate. In addition, FOC-fed mice had higher blood cell counts and less weight loss compared with those on a corn oil diet. This study suggests that FOC could be a beneficial adjunct to chemotherapy, helping to improve patient recovery and reduce side-effects between treatment cycles [136]. Furthermore, it was observed that ω-3 PUFA supplementation can induce vascular remodeling in tumors from rats bearing chemically induced breast cancer before and during docetaxel treatment [137]. Indeed, ω-3 PUFA supplementation increased the microvascularization by 15% and decreased the macrovascularization by 80%, along with a reduced number of large vessels compared with the control group. Once chemotherapy treatment started, ω-3 PUFAs increased the tumor regression induced by docetaxel by 70% [137].

## Clinical trials

A search of ClinicalTrial.gov using “breast cancer” and “ω-3 PUFAs” yields 43 clinical trials, among which 7 (Table 1) were set up to explore the action of ω-3 PUFAs in combination with chemotherapy in patients with breast cancer. Studies differ in terms of the intervention, including the doses and formulation of ω-3 PUFAs. Most of them aim to investigate whether ω-3 PUFAs improve the efficacy of the drug treatment, reducing the side-effects and ameliorating quality of life. In particular, changes in fatigue and pain levels, cognitive functions, and life measures represent the main outcomes of these clinical trials. Although four clinical trials have been completed, only one has published its results. In a randomized double-blind placebo-controlled trial, the authors enrolled 80 breast cancer female patients aged between 30 and 70 years and undergoing treatment with four courses of paclitaxel 175 mg/m^2^. Subjects were divided into two groups: one receiving soft gelatin capsules containing 640 mg of ω-3 PUFAs (54% DHA, 10% EPA) 3 times/day during paclitaxel treatment and 1 month after the end of chemotherapy, and another one supplemented with sunflower soft gelatin capsules as placebo. ω-3 PUFA supplementation demonstrated protective effects against the peripheral neuropathy induced by paclitaxel in patients with breast cancer. In particular, the authors evidenced a reduced incidence of peripheral neuropathy induced by paclitaxel and an increased sural nerve sensory action potential in women taking ω-3 PUFAs compared with the control group, highlighting the neuroprotective effects of ω-3 PUFAs and its role in improving patient outcome [138]. These encouraging results support further exploration of the role of ω-3 PUFAs in the breast cancer setting, which may also yield new insights into preventing the emergence of chemoresistance.

**Table 1:** Clinical trials using ω-3 PUFAs and chemotherapeutic drugs in patients with breast cancer registered at https://clinicaltrials.gov (accessed on 16 June 2024)

Study	Phase	State	Intervention/treatment	Eligibility criteria, primary outcome, and purpose	Results
NCT02352779	N/A	Completed with results	-Low-dose of ω-3 PUFAs orally (PO) twice daily (BID) and placebo PO BID for 6 weeks -High-dose ω-3 PUFA supplementation PO BID for 6 weeks -Placebo	Eligibility criteria: women with confirmed diagnosis of breast cancer and who had undergone some type or combination of standard adjuvant treatment having cancer-related fatigue. Primary outcome: cancer-related fatigue and Multidimensional Fatigue Symptom Inventory-Short Form. Purpose: to investigate the effects of ω-3 PUFAs in reducing cancer-related fatigue in BC survivors	[138]
NCT01821833	N/A	Completed	– ω-3 PUFA capsules orally beginning 1 week prior to paclitaxel treatment – Placebo prior to paclitaxel treatment	Eligibility criteria: patients having a diagnosis of breast cancer or ovarian cancer who are scheduled to receive weekly paclitaxel at 70–90 mg/m^2^ for a minimum of 2 months; ECOG performance status of 0, 1, or 2. Primary outcome: mean severity of pain, incidence of pain, or relief. Purpose: to determine whether ω-3 PUFAs reduce pain in patients with breast or ovarian cancer receiving paclitaxel	
NCT05331807	Early phase I	Recruiting	– Two capsules of ω-3 PUFAs daily – One capsule of vitamin D3 weekly – Two capsules of ω-3 PUFAs and one capsule of vitamin D3 weekly	Eligibility criteria: women with newly diagnosed stage I–III BC who will receive treatment with adriamycin and cytoxan for a total of four cycles. Primary outcome: changes in body mass index, body weight, muscle mass status, nutritional status condition, total scale, and single-item measures scores, blood inflammatory markers. Purpose: to assess the effect of combined ω-3 PUFAs and vitamin D supplementation on nutritional status, quality of life, and blood inflammatory markers among women with BC undergoing chemotherapy treatment in the Gaza Strip, Palestine	
NCT01823991	Early phase I	Completed	– COGNUTRIN (40% polyphenolics, 12.5% anthocyanins from blueberries and omega-3) for 3 months – Placebo	Eligibility criteria: women with stage II–IIIA breast cancer who have completed adjuvant treatment with anthracyclines and taxanes + or − radiation therapy within the past 6 months and do not show any evidence of dementia. Primary outcome: cognitive function score changes. Purpose: to investigate the safety and the influence of COGNUTRIN on cognitive performance in BC survivors following chemotherapy	
NCT01049295	4	NA	– 640 mg oral oil fish pearls (54% DHA, 10% EPA) three times a day – Placebo	Eligibility criteria: women with invasive breast cancer, not receiving any form of supplementations and oil fish. Primary outcome: serum level of ω-3 PUFAs before chemotherapy with taxanes and after 3 months. Purpose: to evaluate the effects of ω-3 PUFAs on taxane-induced neuropathy and inflammation in patients with invasive BC	
NCT02795572	2	Terminated	– Daily dose of vitamin D 2000 IU, vitamin B6 100 mg, vitamin B12 100 mcg, and ω-3 PUFAs 2700 mg – Placebo	Eligibility criteria: women aged between 18 and 70 with invasive breast carcinoma receiving docetaxel. Primary outcome: chemotherapy induced peripheral neuropathy assessment. Purpose: to evaluate the effects of nutraceuticals, including ω-3 PUFAs, in patients treated with docetaxel as neoadjuvant or adjuvant BC therapy	
NCT02517502	Early phase 1	Completed	– 400 mg capsules of DHA daily – Placebo	Eligibility criteria: women aged 45–70 years with stage I–III invasive breast cancer who will start neoadjuvant chemotherapy. Primary outcome: evaluation of the proportion of eligible subjects agreeing to participate in the study and successfully complete the cognitive assessments. Number of subjects reporting serious adverse effects. Purpose: to investigate whether DHA administered prior to and in combination with neoadjuvant chemotherapy is able to prevent or reduce the cognitive dysfunction induced by chemotherapy	

N/A, not applicable

## Challenges for co-delivery of nanomedicines based on the combination of ω-3 PUFAs and chemotherapeutic drugs

Although natural compounds, including ω-3 PUFAs, have demonstrated promising actions as adjuvant molecules in breast cancer prevention and treatment, they are characterized by reduced solubility, stability, and delivery efficacy, which may limit their utilization in the clinical setting. Nanocarriers for drug delivery represent good tools to overcome these obstacles and develop new therapeutic strategies for breast cancer treatment. In particular, tailored nanocarriers loaded with chemotherapeutic drugs and natural compounds have been shown to improve chemotherapy efficacy and reduce the size effects in different types of cancer [139]. Indeed, these delivery systems can target specific molecules of chemoresistant cells, enhancing the drug accumulation and antitumoral effects (active targeting) [140]. Moreover, targeted nanocarriers allow a more selective biodistribution of a drug into the tumor site, reducing damage to healthy tissue and off-target effects [141]. Thirdly, employing nanosystems that transport antineoplastic agents can circumvent drug resistance mechanisms, particularly those associated with efflux pumps, since nanocarriers are not substrates for these pumps [142]. In TNBC, numerous targets, including trophoblast cell-surface antigen 2 (Trop-2), protein tyrosine kinase 7 (PTK7) receptor, ephrin receptor-4, folic acid, and neuropilin-1, have been exploited [143]. Targeting of nanoparticles is attracting much attention, especially for the treatment of TNBC, owing to the lack of expression of the molecular targets for breast cancer treatment in this subtype of neoplasia [143]. Regarding ω-3 PUFAs, the effects of DHA combined with doxorubicin have been investigated in nanostructured lipid carriers, without active targeting techniques. Mussi and collaborators showed that encapsulation of DHA with doxorubicin in their delivery system increased the chemotherapeutic drug efficacy in an adriamycin (ADR)-resistant MCF-7/ADR breast cancer cell line [144]. Thus, targeting the nanocarrier loaded with ω-3 PUFAs and chemotherapeutic drug with molecules, including peptides or antibodies, able to selectively bind to breast cancer cells, might improve the efficacy of chemotherapy, potentially reducing its side effects in healthy tissues.

## Conclusions

It is universally recognized that conventional chemotherapy has debilitating adverse effects in patients with breast cancer, profoundly impacting their well-being. On the other hand, the occurrence of chemoresistant tumors is the main contributor to cancer-related mortality. Hence, it becomes imperative to devise strategies that reduce chemotherapy-associated toxicities while maximizing the efficacy of existing chemotherapy drugs in the curative therapeutic setting. ω-3 PUFAs have been documented as promising adjuvant molecules in breast cancer. In vitro and in vivo studies have documented the role of ω-3 PUFAs as promising adjuvant molecules in breast cancer. Furthermore, clinical studies are currently underway to assess the effects of ω-3 PUFAs in alleviating the side effects observed in patients undergoing chemotherapy. The collective mechanisms through which ω-3 PUFAs may function as both chemotherapeutic and chemopreventive agents can be summarized into three main approaches: directly exerting tumoricidal activities via several signaling pathways, reversing chemoresistance-related mechanisms, and mitigating the toxicity induced by chemotherapeutic drugs. However, it is imperative to underscore that these natural products are not meant to replace traditional cancer treatments, such as chemotherapy. Rather, they should augment existing therapies and be integrated into a comprehensive approach tailored to the specific needs of each patient. In this context, co-delivering ω-3 PUFAs or their derivatives with chemotherapeutic drugs using functionalized nanoparticles may represent a promising drug delivery platform for precise cancer treatment. Future and rigorous scientific investigation are essential to fully elucidate the precise mechanisms of action of ω-3 PUFAs, determine optimal dosages, and identify effective combinations with antitumor therapeutic strategies. This knowledge will be crucial in harnessing their full potential in cancer treatment, thereby providing hope and improved outcomes for patients with breast cancer globally.

## Data Availability

Not applicable.

## Abbreviations

ABC: ATP-binding cassette
AKT: Protein kinase
ALDH: Aldehyde dehydrogenase
AMPK: AMP-activated protein kinase
ATM: Ataxia telangiectasia mutated
BCRP: Breast cancer resistance protein
BID: BH3 interacting domain death agonist
CAAs: Cancer-associated adipocytes
CAFs: Cancer-associated fibroblasts
cAMP: Cyclic adenosine 3′,5′-monophosphate
CPTI: Carnitine palmitoyltransferase I
CSF: Colony-stimulating factor
CXCL: C–X–C motif chemokine ligand
DHA: Docosahexaenoic acid
DSBs: Double-strand breaks
ECs: Endothelial cells
ECM: Extracellular matrix
EGFR: Epidermal growth factor receptor
EMT: Epithelial-to-mesenchymal transition
EPA: Eicosapentaenoic acid
ERBB2: Epidermal growth factor 2
EVs: Extracellular vesicles
FASN: Fatty acid synthase
FOC: Fish oil concentrate
HIF: Hypoxia-inducible factor
HR: Hormone receptors
HR: Homologous recombination
IL: Interleukin
JAK: Janus kinase
LDL: Low-density lipoproteins
LKB1: Liver kinase B1
MCP: Chemoattractant protein
MDR: Multidrug resistance
MMP: Matrix metalloproteinase
MRP1: Multidrug-resistant protein-1
MSCs: Mesenchymal stem cells
mTOR: Mammalian target of rapamycin
NF-κB: Nuclear factor kappa B
NHEJ: Nonhomologous end-joining
PDX: Patient-derived xenografts
P-gp: P-glycoprotein
PKA: Protein kinase A
PPAR: Peroxisome proliferator-activated receptor
PTK7: Protein tyrosine kinase 7
ROS: Reactive oxygen species
RTKs: Receptor tyrosine kinases
SCD1: Stearoyl-CoA desaturase
SDC-1: Tumor suppressor syndecan-1
STAT: Signal transducer and activator of transcription
TAMs: Tumor-associated macrophages
TGF: Transforming growth factor
TNBC: Triple-negative breast cancer
TNF: Tumor necrosis factor
TNFR1: TNF receptor 1
TRAD: TNFR1-associated death domain protein
TROP-2: Trophoblast cell-surface antigen 2
VEGFR: Vascular endothelial growth factor receptor
ω-3 PUFAs: Omega-3 polyunsaturated fatty acids
